# Detecting early‐warning biomarkers associated with heart‐exosome genetic‐signature for acute myocardial infarction: A source‐tracking study of exosome

**DOI:** 10.1111/jcmm.18334

**Published:** 2024-04-25

**Authors:** Xiaojun Jin, Weifeng Xu, Qiaoping Wu, Chen Huang, Yongfei Song, Jiangfang Lian

**Affiliations:** ^1^ The Affiliated Lihuili Hospital of Ningbo University Health Science Center, Ningbo University Ningbo Zhejiang China; ^2^ Department of Genetics The First Affiliated Hospital of Xi'an Jiaotong University Xi'an Shaanxi China

**Keywords:** clustering, early‐warning, heart‐exosomes, prediction, source‐tracking

## Abstract

The genetic information of plasma total‐exosomes originating from tissues have already proven useful to assess the severity of coronary artery diseases (CAD). However, plasma total‐exosomes include multiple sub‐populations secreted by various tissues. Only analysing the genetic information of plasma total‐exosomes is perturbed by exosomes derived from other organs except the heart. We aim to detect early‐warning biomarkers associated with heart‐exosome genetic‐signatures for acute myocardial infarction (AMI) by a source‐tracking analysis of plasma exosome. The source‐tracking of AMI plasma total‐exosomes was implemented by deconvolution algorithm. The final early‐warning biomarkers associated with heart‐exosome genetic‐signatures for AMI was identified by integration with single‐cell sequencing, weighted gene correction network and machine learning analyses. The correlation between biomarkers and clinical indicators was validated in impatient cohort. A nomogram was generated using early‐warning biomarkers for predicting the CAD progression. The molecular subtypes landscape of AMI was detected by consensus clustering. A higher fraction of exosomes derived from spleen and blood cells was revealed in plasma exosomes, while a lower fraction of heart‐exosomes was detected. The gene ontology revealed that heart‐exosomes genetic‐signatures was associated with the heart development, cardiac function and cardiac response to stress. We ultimately identified three genes associated with heart‐exosomes defining early‐warning biomarkers for AMI. The early‐warning biomarkers mediated molecular clusters presented heterogeneous metabolism preference in AMI. Our study introduced three early‐warning biomarkers associated with heart‐exosome genetic‐signatures, which reflected the genetic information of heart‐exosomes carrying AMI signals and provided new insights for exosomes research in CAD progression and prevention.

## 1 INTRODUCTION

Coronary artery disease (CAD), including stable CAD (SCAD) and acute myocardial infarction (AMI), is a frequent cardiovascular disorder. If left untreated, SCAD could progress to AMI. When myocardial remodelling begins, irreversible myocardial‐tissue injury occurs due to obstructive ischemia after AMI.^1^ In clinical practice, well‐established heart biomarkers are currently used for diagnosing AMI. Unfortunately, no effective methods currently exist for providing early warnings for AMI occurrence. Therefore, defining biomarkers associated with CAD severity and identifying individuals at risk of AMI prior to overt disease becoming apparent using risk biomarkers will be enable strategies for early warnings and early treatment to prevent progression to AMI.

Exosomes (measuring 30–150 nm in diameter), which are lipid belayer‐enclosed vesicles, are released by living cells into the extracellular space in response to hypoxia and inflammation.^2^, ^3^ They act as intracellular messengers, plasma through all body fluids, and carry a range of biologically active cargo, including proteins and genetic materials, from the host cells. This has been shown to indicate pathophysiology or physiological changes and has emerged as a non‐invasive liquid biopsy strategy for cardiovascular diseases.^4^ Altered micro ribonucleic acid (RNA) and messenger RNA (mRNA) in plasma total exosomes from various tissues have proven to be useful for assessing the extent of disease among different stages of CAD and play an essential role in biological processes from SCAD to AMI.^2^, ^5^, ^6^ However, plasma total exosomes are heterogeneous vesicles comprising multiple sub‐populations secreted by various tissues, and the genetic networks between these sub‐populations and different organs are complex.^7^ Only analysing the genetic information of plasma total exosomes is likely to be complicated by exosomes derived from other organs except for the heart. Therefore, source analysis of plasma total exosomes RNAs and identification of early‐warning biomarkers associated with heart exosome genetic signatures are essential for accurately predicting early warnings and preventing later AMI occurrences. In addition, changes in expression patterns of these heart exosome genetic signatures also provide precise information regarding changes in the pathophysiological states of the heart tissue.

**FIGURE 1:**
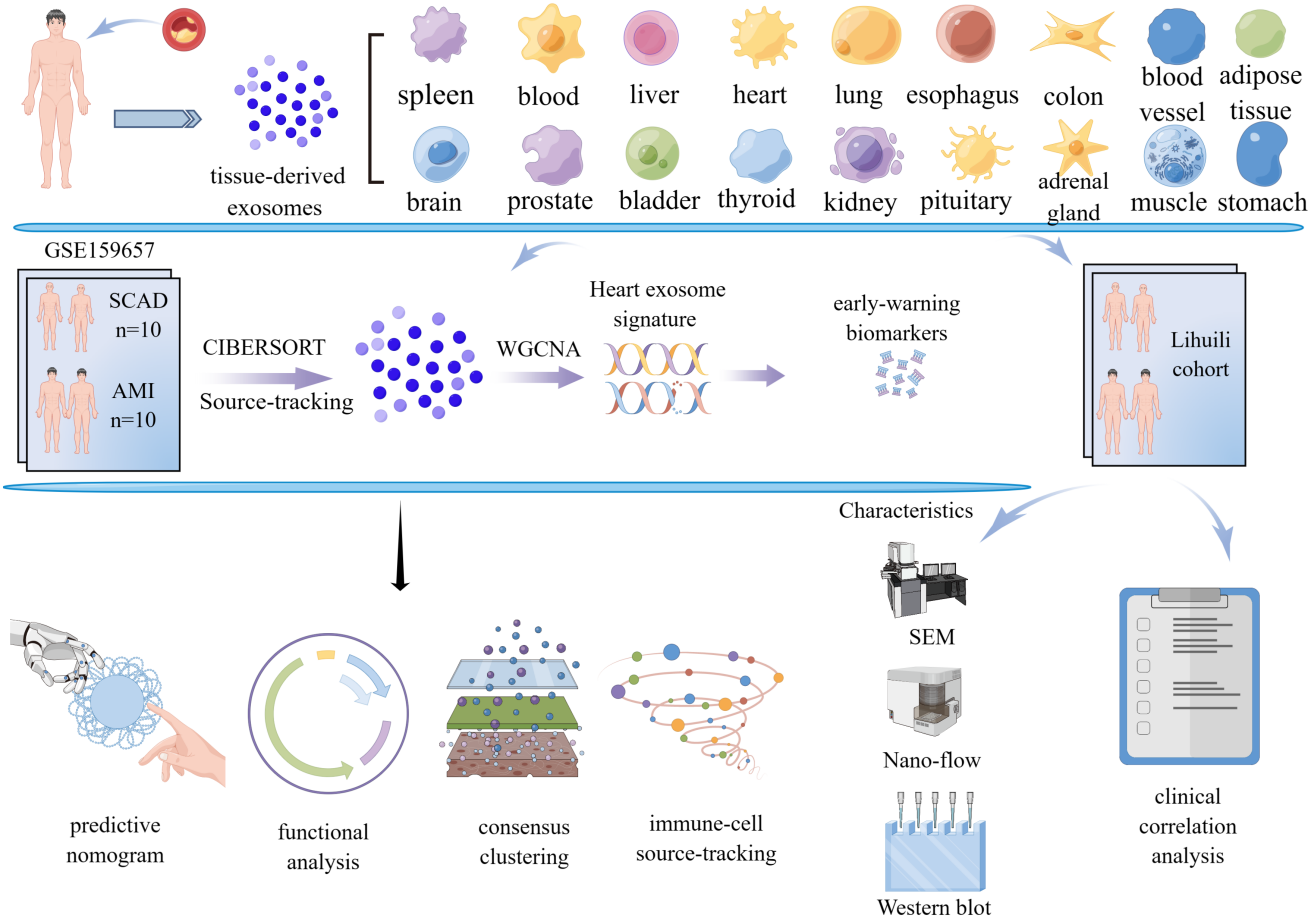
A schematic showing study procedures.

Our objective here was to profile the tracking analysis for the origins of plasma total exosomes at a tissue level, using the exosomal RNA sequencing approach, to find early‐warning biomarkers that had genetic connections between plasma exosomes and the heart to help identify individuals at high risk of AMI. The mechanisms linking AMI to early‐warning biomarkers were further explored. A schematic of study procedures was shown in Figure 1.

## 2 METHODS

### 2.1 Data acquisition and processing

The exosomal RNA sequencing data of plasma samples comprising eight patients with SCAD and 10 patients with AMI were obtained from Gene Expression Omnibus under the accession number GSE159657. The data were converted from raw counts format to the fractions per kilo base format, and log_2_‐transformation and mean normalization were applied. Exosomally differentially expressed genes (DEGs) with the criteria of logFC >0.2 and *p* < 0.05 between the groups were calculated using the R limma package, followed by visualization with volcano plots.

### 2.2 Source‐tracking of exosomes and the construction of weighted gene co‐expression networks

The source‐tracking of plasma total exosomes was implemented by estimating relative subsets of the RNA transcript (CIBERSORT) algorithm, which was consistent with previously published studies.^8^, ^9^ Briefly, the tissue‐specific genes (TSGs) of the spleen, whole blood cells, oesophagus, colon, blood vessel, adipose tissue, brain, prostate, bladder, thyroid, kidney, liver, pituitary, heart, lung, adrenal gland, muscle and stomach were retrieved from the published study^9^ and the Tissue‐specific Gene DataBase in cancer website (Table S1). The TSG expression levels were obtained from the Genotype‐Tissue Expression portal database. The signature matrix was created by the CIBERSORTx online tool based on the TSG expression levels, and the relative fractions of different tissue‐derived exosomes were subsequently inferred by the CIBERSORT deconvolution algorithm based on the signature matrix. The weighted gene co‐expression network analysis (WGCNA) was broadly adopted to identify a specific clinical trial‐related gene set. The WGCNA approach was applied to GSE159657 expression data using the R WGCNA package to identify the correlation between gene expression and the relative fraction of heart exosomes and infer the heart exosomes‐related genetic signature in AMI. Outlier samples were assessed via hierarchical clustering, followed by scale‐free network construction. An adjacent matrix was constructed using the selected soft threshold, which was obtained from the pick soft function analysis and converted into a corresponding topological overlap matrix (TOM). The gene network was hierarchically clustered using 1–TOM as the distance measure to identify the gene groups (module eigengenes, ME) whose expression varied across clinical traits. Modules whose correlations of ME exceeded a threshold of 0.75 were merged. Correlations between modules and clinical traits were identified using Pearson correlation analysis.

### 2.3 Single‐cell (sc) RNA (scRNA) sequencing data processing

The scRNA‐sequencing data of human left‐ventricle tissue, comprising two normal and six AMI samples, were obtained from the GSE145154 dataset and processed using the R Seurat package. Four Seurat objects were combined into a merged object, and the fractions of mitochondrial genes were calculated for each cell by the PercentFeatureSet function. Cells with fewer than 200 genes per cell, cells with more than 2500 genes per cell, and cells that had more than 20% unique molecular identifiers from mitochondrial genes were filtered out. The filtered count matrix was log‐transformed, followed by the identification of the top 2000 most variably expressed genes through the FindVariableFeatures function. Each gene expression was scaled, and principal components analysis was performed using the top 2000 variable genes to reduce the initial dimensionality prior to further reduction through t‐distributed stochastic neighbour embedding (t‐SNE). Clustering was performed with the resolution parameter set to 1 and the dim set to 30 using the FindCluster function. The FindMarker function was then used to identify the top 10 highly expressed genes of each cluster for cell‐type annotation. Finally, the FindMarker function was used to identify sc DEGs for cardiomyocyte (CM) populations between the groups.

### 2.4 Machine learning

The least absolute shrinkage and selection operator‐logistic regression, random forest and support vector machine recursive feature elimination were implemented in thus study. The intersection genes between the DEGs, sc DEGs, and heart exosome signature yielded candidate early‐warning biomarkers in AMI. The integration of the three machine learning algorithms was used here to determine which of these candidate early‐warning biomarkers were key for maximizing the discriminative power. The intersection of gene expression levels from machine learning algorithms across exosomes was visualized using boxplots.

### 2.5 Construction of a predictive nomogram

The rms package was used to construct a nomogram based on the intersecting gene expression patterns to predict the probability of AMI occurrence among patients with SCAD. Calibration plots compared the predicted probability calculated using the nomogram with the actual outcome and assessed the prediction model's accuracy.

### 2.6 Functional analysis and its correlation with early‐warning biomarkers

The single‐sample gene set enrichment analysis (ssGSEA) algorithm was performed to assign enrichment scores of molecular pathways to each sample in the gene set variation analysis package, with ‘c2.cp.kegg.v7.0.symbols.gmt’ gene set derived from the Molecular Signatures Database. Each enrichment score represented the extent to which the molecular functions in the gene set were relatively up‐ or down‐regulated within a sample. Spearman's correlations were used to define the correlations between enrichment scores and expression profiles of early‐warning biomarkers. In addition, the activation or inhibition‐related pathway that could be promoted by RPL23, PKIG and OST4 in AMI samples was complemented by gene set enrichment analysis methods with ‘c2.cp.kegg.v7.0.symbols.gmt’ gene set. The significant pathways were filtered by the normalized enrichment scores (NES) >1 and nominal *p* < 0.05.

### 2.7 Identification of molecular subtypes based on the early‐warning biomarkers in AMI

AMI samples were grouped based on the expression profiles of early‐warning biomarkers via the k‐means clustering analysis, with the *k*‐value set between 2 and 5. The optimal number of clusters was determined from the consensus matrix and cumulative distribution function curves. Additionally, 115 metabolism‐related gene signatures were collected from previous studies.^10^ The ssGSEA was performed to evaluate the enrichment of the Kyoto Encyclopaedia of Genes and Genomes (KEGG) molecular and metabolism pathways in the subtypes of patients with AMI.

### 2.8 Human studies and the isolation of plasma exosomes

The Ethics Committee of The Affiliated Lihuili Hospital of Ningbo University approved the human studies. Informed consent was obtained from all participants. Plasma samples were obtained from seven patients with CAD within 48 h of hospitalization prior to coronary intervention. Exosome were isolated from each sample's equivalent volume of plasma following established protocols. The plasma samples were centrifuged at 10,000*g* for 1 h to remove cell debris. Following this, 500 μL of cell debris‐free plasma was added to 400 μL Umibio exosome precipitation solution A to remove plasma proteins, followed by the addition of 120 μL solution B and centrifugation at 12,000 rpm/min for 15 min. The preliminary exosome particles were then purified by an exosome purification filter at 5600 rpm/min for 20 min. The final exosome products were dissolved with 50 μL of phosphate‐buffered saline (PBS) and applied for downstream RNA level analyses. As for western blot assay, exosome from 500 μL of cell debris‐free plasma was isolated by Exosupur size exclusion column following the instruction manual (Echobiotech, China).

### 2.9 Exosome lysis and real‐time quantitative polymerase chain reaction (qPCR)

Each sample's 5 μL exosome product was added to 5 μL of lysis buffer, followed by lysis at 37°C for 10 min and 75°C for 10 min. Then, the lysis product was reverse‐transcribed using the reverse transcription kit. A real‐time qPCR was performed using 1 μL of complementary deoxyribonucleic acid and SYBR qPCR Master Mix Kit (Vazyme) per the manufacturer's instructions. The relative differences in messenger mRNAs levels were measured by the 2‐ΔΔCt method. The gene primer sequences are presented in Table S2.

### 2.10 Western blotting

The human CM line AC16 was obtained from the cell bank of the Chinese Academy of Science (Shanghai, China). The bicinchoninic acid assay was used to determine the total protein quantity from AC16 cells and exosomes. The proteins were separated on sodium dodecyl sulphate gel and transferred onto the polyvinylidene fluoride membranes. The proteins on the membranes were blocked and probed with primary antibodies against rabbit anti‐CD9 (Proteintech), rabbit anti‐CD81 (Proteintech) and rabbit anti‐Calnexin (Proteintech) overnight at 4°C, followed by incubation with the secondary antibodies against HRP‐lablled sheep anti‐rabbit immunoglobulin (Ig) G (Proteintech) for 1 h at room temperature. Enhanced chemiluminescence (Proteintech) was used to visualize the bands.

### 2.11 Nano‐flow cytometry analysis of exosomes

The size ranges of the resultant exosomes were measured by nano‐flow cytometry (NanoFCM). Furthermore, 30 μL of exosomes were added to 20 μL of fluorescein isothiocyanate (FITC)‐labelled mouse anti‐human CD9 (BD) and FITC‐labelled mouse anti‐human CD81 (BD) antibodies followed by incubation at room temperature for 30 min. With the exception of the primary antibody, other homogenization treatments were set as negative controls. The mixture was added to 1 mL PBS, followed by centrifugation at 110,000*g* for 70 min. The exosomes were resuspended again in 50 μL of PBS and subsequently detected.

### 2.12 Statistical analysis

The R software (version 4.2.1) was used to perform data analyses. Pairwise comparisons were made using Student's *t*‐test for normally distributed variables or the Wilcoxon rank‐sum test for non‐normally distributed variables. Statistical significance was set at *p* < 0.05.

## 3 RESULTS

### 3.1 Source‐tracking of plasma exosome reflects the heart exosome proportion and gene signature

**FIGURE 2:**
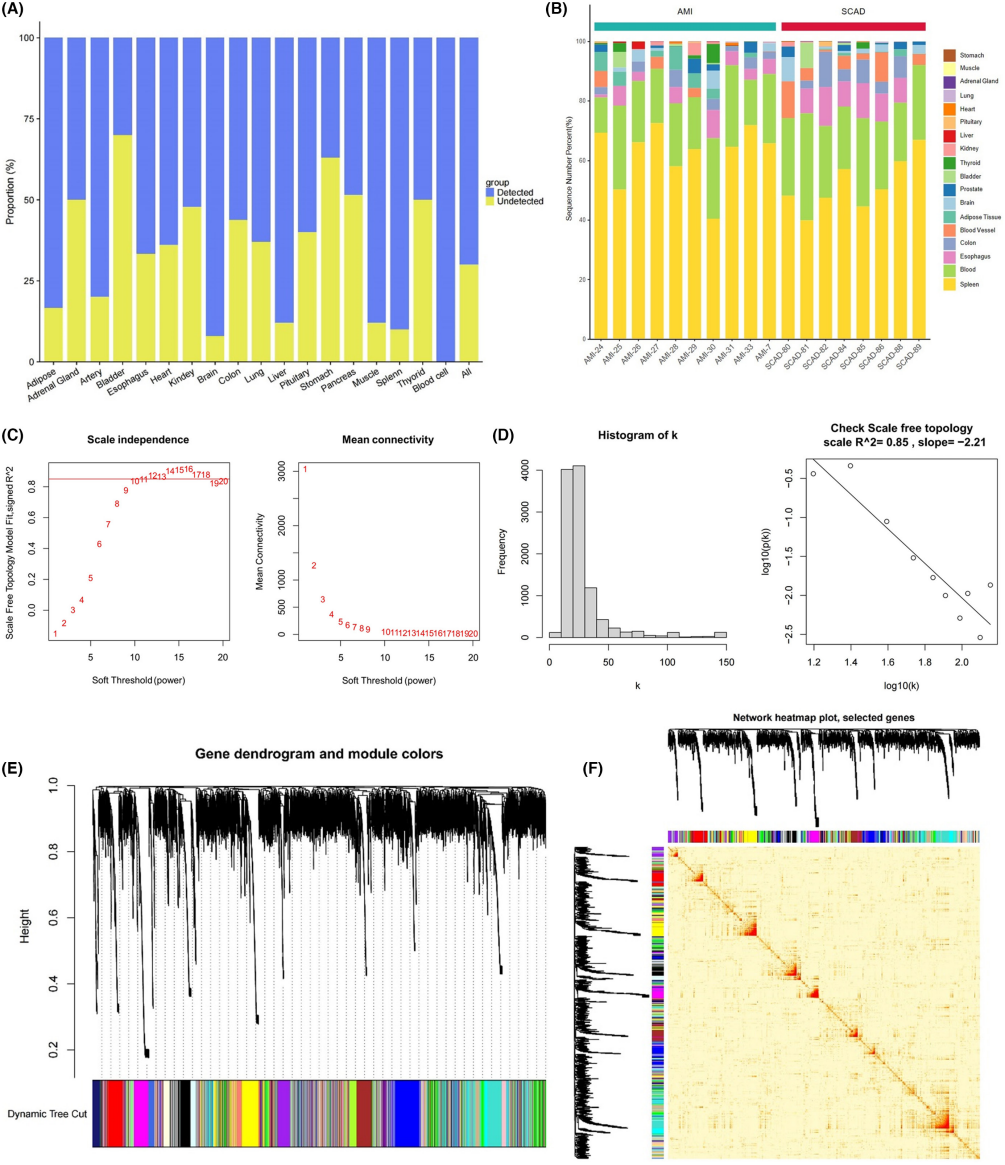
The source‐tracking of exosome and weighted gene co‐expression network analyses. (A) The number of tissue‐specific genes from different tissues across tissues detected in plasma exosomes. Blue, detected; Yellow, non‐detected. (B) The deconvoluted tissue‐specific proportion analysis of the plasma exosome sources. (C, D) The schematic representation of a scale‐free index (C) and mean connectivity (D) under different soft‐threshold powers. (E) Cluster dendrogram demonstrating co‐expression developed by average linkage hierarchical clustering and dynamic hierarchical cut. (F) The Topological Overlap Matrix.

**FIGURE 3:**
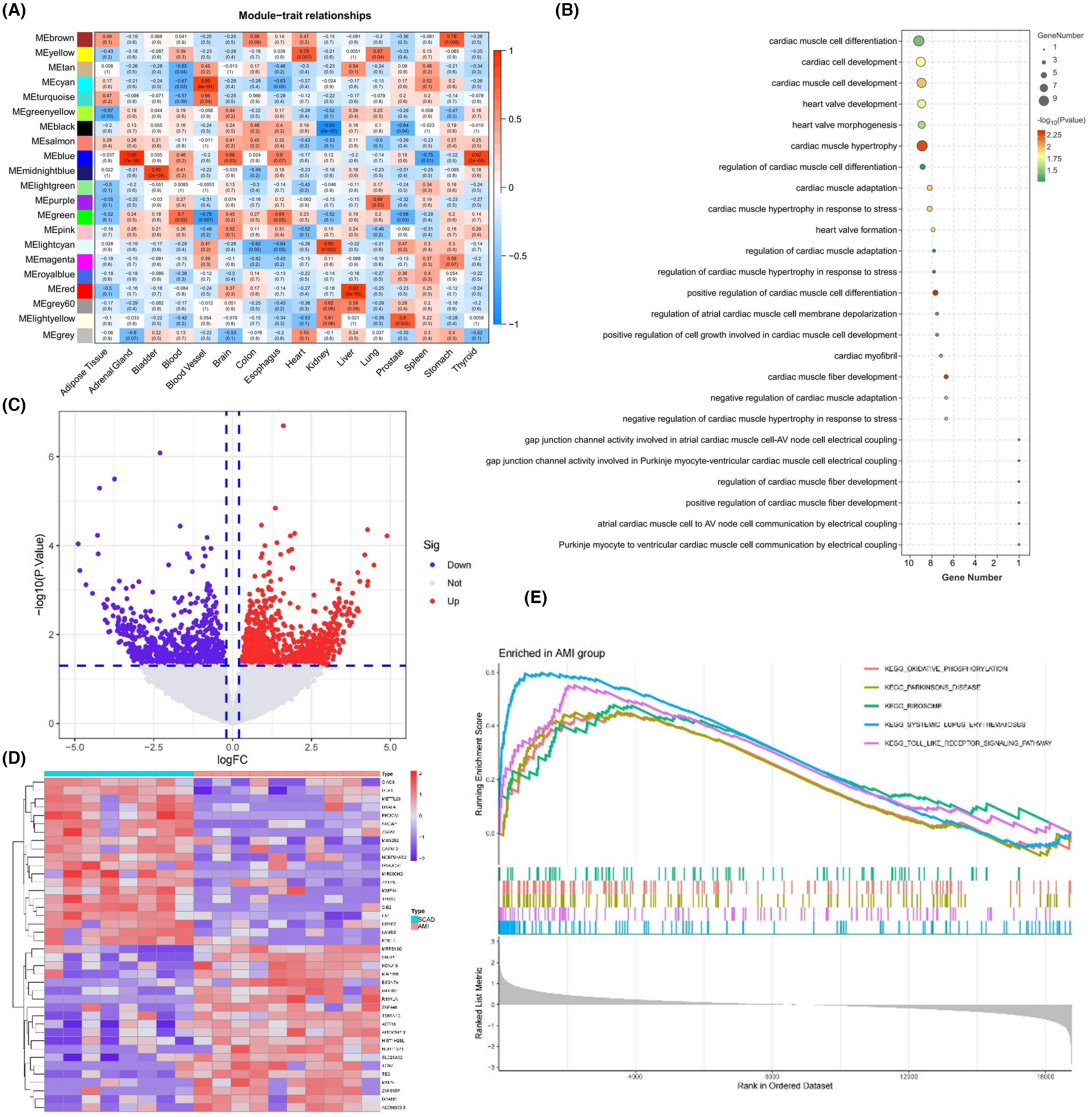
Heart exosome genetic signature enrichment and differential expression analyses. (A) The heatmap of modules‐trait relationship. (B) The bubble plot of the Gene Ontology analyses for heart exosome genetic signatures. (C) The volcano plot of differential expression genes. (D) The heatmap of the top 40 differentially expressed genes between acute myocardial infarction (AMI) and stable coronary artery disease (SCAD) groups. (E) The pathway enrichment in the AMI group from the gene set enrichment analysis.

About 70% of TSG were detected in the plasma exosome samples of patients with CAD (Figure 2A), suggesting that these exosomes were enriched with diverse TSG. The CIBERSORT algorithm estimated the relative abundance of various exosome sub‐populations in plasma exosomes, which showed a moderately higher fraction of exosomes derived from the spleen and blood cells and a lower fraction of heart exosomes (Figure 2B). The WGCNA method was applied to the RNA sequencing data of AMI exosomes to explore the association between gene expression signatures with the relative fraction of exosome sub‐populations from various tissues and identify genetic signatures associated with heart exosomes. A soft‐threshold power was set to 12, at which the scale‐free topology fit index reached 0.9 (Figure 2C,D). Twenty‐one gene modules were identified by WGCNA via average‐linkage hierarchical clustering and dynamic tree clipping (Figure 2E,F). The yellow module showed the strongest association with the relative fraction of heart exosomes (correlation = 0.79, *p* = 0.0079, Figure 3A). A total of 779 genes in the yellow module were denoted as heart exosome‐associated genes, followed by construction with enrichment analyses.

In addition to membrane‐bounded organelles and membrane‐enclosed lumens and cell parts in the cellular component categories, the Gene Ontology (GO) analysis of these heart exosome‐associated genes revealed significant enrichment in the biological process categories associated with the terms of heart development, cardiac function and cardiac response to stress, including positive regulation of cardiac muscle cell differentiation, cardiac muscle fibre development, cardiac muscle cell development, cardiac muscle hypertrophy in response to stress and heart valve development (Figure 3B, Table S3).

DEG analyses from exosomal RNA sequencing identified 752 up‐regulated and 699 down‐regulated genes in the AMI group compared with the SCAD group (Figure 3C,D). According to the GSEA pathway analyses, oxidative phosphorylation, ribosomes, systemic lupus erythematosus and the toll‐like receptor signalling pathway were modulated in response to AMI (Figure 3E), reflecting that the plasma exosomes actively participated in these pathways during CAD progression.

### 3.2 Differential expression analyses of CMs with ScRNA‐sequencing data processing

**FIGURE 4:**
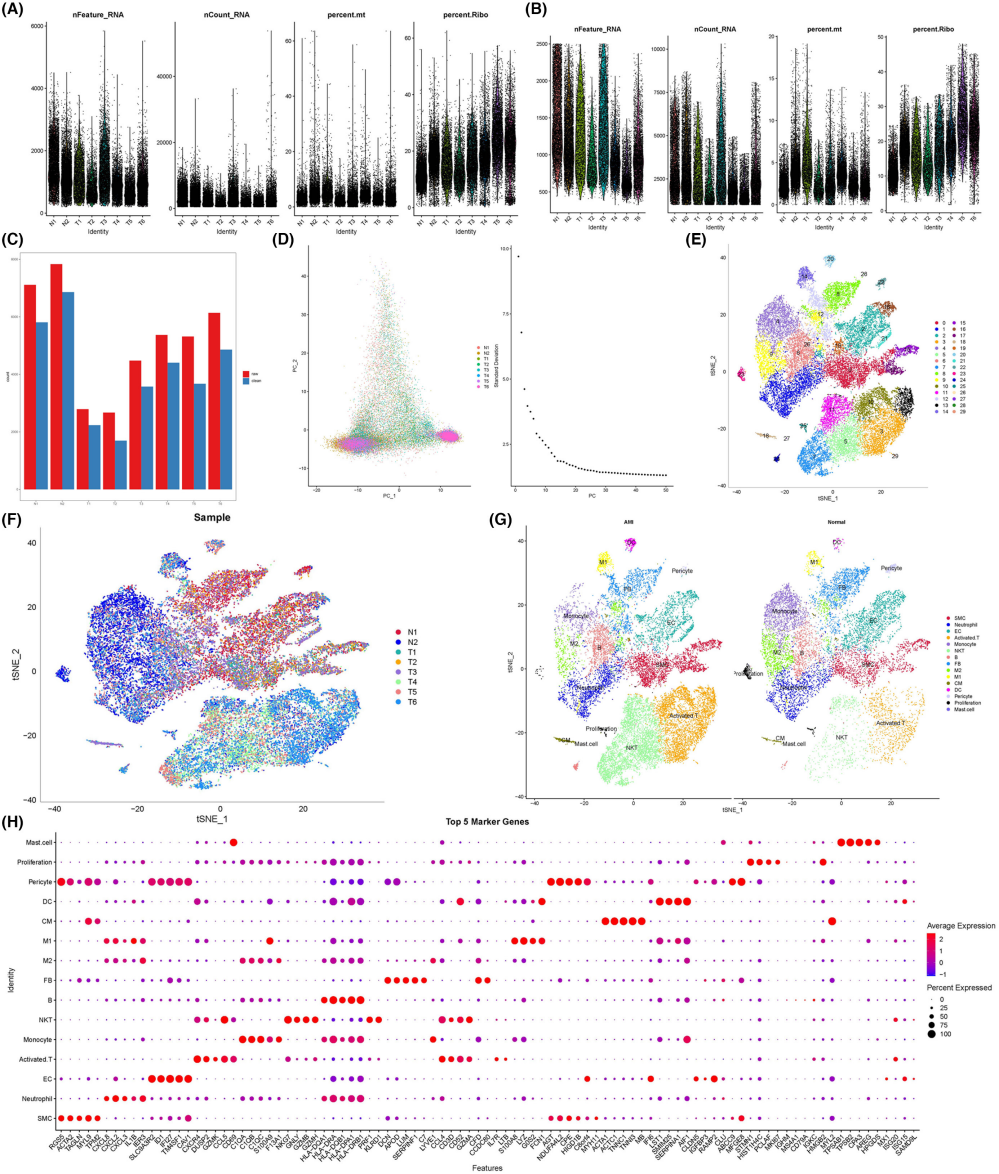
The single‐cell ribonucleic acid (RNA) sequencing data analysis. (A, B) Characteristics of the RNA content and mitochondrial genes in single cells before (A) and after (B) quality control. (C) The principal component analysis of every single cell across samples. (D) The optimal parameter selection of dimensionality reduction. (E, F) t‐distributed stochastic neighbour embedding (t‐SNE) the plots of 30 clusters (E) of different sample types (F). (G) Overview of the cell types in different samples based on the t‐SNE plot. (H) Dot plot of the top 10 marker genes enriched in each cell type.

Given the dominant physiological and pathological characteristics of AMI being caused by CM apoptosis and necrosis and the alteration of DEGs landscape at the sc level before and after AMI, scRNA‐sequencing data was processed. After filtering out cells with a low gene count and a high mitochondrial transcript ratio, 10,000 cells passed quality control (Figure 4A–C). Principal component analysis was adopted for dimensionality reduction, and the optimal parameter of dimensionality reduction was evaluated by elbow plot (Figure 4D). We identified 30 clusters which were visualized via a t‐SNE plot by clustering the 10,000 cells in the atlas (Figure 4E). An overview of the single cell within each sample is presented in Figure 4F. These identified clusters were annotated into 15 distinct cell types based on the canonical genes of each cluster (Figure 4G). Marker genes enriched in each cell type are presented in Figure 4H. We identified 1400 sc DEGs for CM populations between the groups.

### 3.3 Multiple machine learning present oligosaccharyltransferase complex subunit 4 (*OST4*), ribosomal protein L23 (*RPL23*), and yclic adenosine monophosphate‐dependent protein kinase inhibitor gamma (*PKIG*)

**FIGURE 5:**
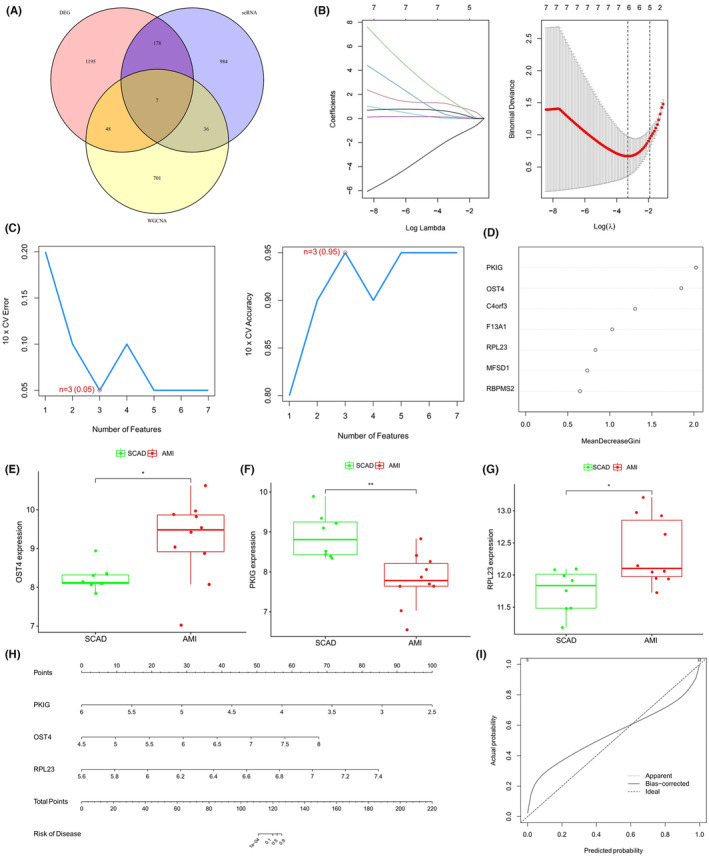
The detection of early‐warning biomarkers and construction of the predictive nomogram. (A) The intersection of differentially expressed genes (DEGs), the weighted gene co‐expression network analysis module genes and single‐cell DEGs. (B) Coefficient changes of different features under different lambda value (left) and feature selection by cross‐validation based on the minimum lambda value (right). (C) The candidate early‐warning biomarkers identified by the support vector machine recursive feature elimination algorithm with the lowest error (left) and rate and the highest accuracy (right). (D) The ranking of candidate biomarkers based on the importance under the random forest classifier. (E–G) Comparison of the gene expression levels of oligosaccharyltransferase complex subunit 4 (E), cyclic adenosine monophosphate‐dependent protein kinase inhibitor gamma (F) and ribosomal protein L23 (G) between groups. (H) The nomogram for predicting the risk of acute myocardial infarction occurrence. (I) Calibration curve for the nomogram validation.

Seven early‐warning biomarkers were identified based on the intersection of the genes identified by exosomal DEGs, the genes identified by WGCNA, and the genes identified by sc DEGs (Figure 5A). Three key early‐warning biomarkers, including *OST4*, *PKIG* and *RPL23*, were screened by the intersection results of diverse machine learning algorithms (Figure 5B–D). Of these biomarkers, *OST4* and *RPL23* were significantly up‐regulated in the AMI samples compared with the SCAD samples, whereas significant *PKIG* down‐regulation was observed (Figure 5E–G).

### 3.4 Early‐warning biomarker expression pattern presents a predictive nomogram reflecting the risk of AMI occurrence

The nomogram, constructed after integrating the *OST4*, *PKIG* and *RPL23* gene expression patterns for quantitatively predicting the probability of AMI occurrence in patients with SCAD patients, was constructed (Figure 5H), demonstrated a marked agreement between predicted and actual outcomes in the calibration plots (Figure 5I).

### 3.5 Exosome characteristics and high *RPL23* and *OST4* levels predict adverse cardiovascular events

**FIGURE 6:**
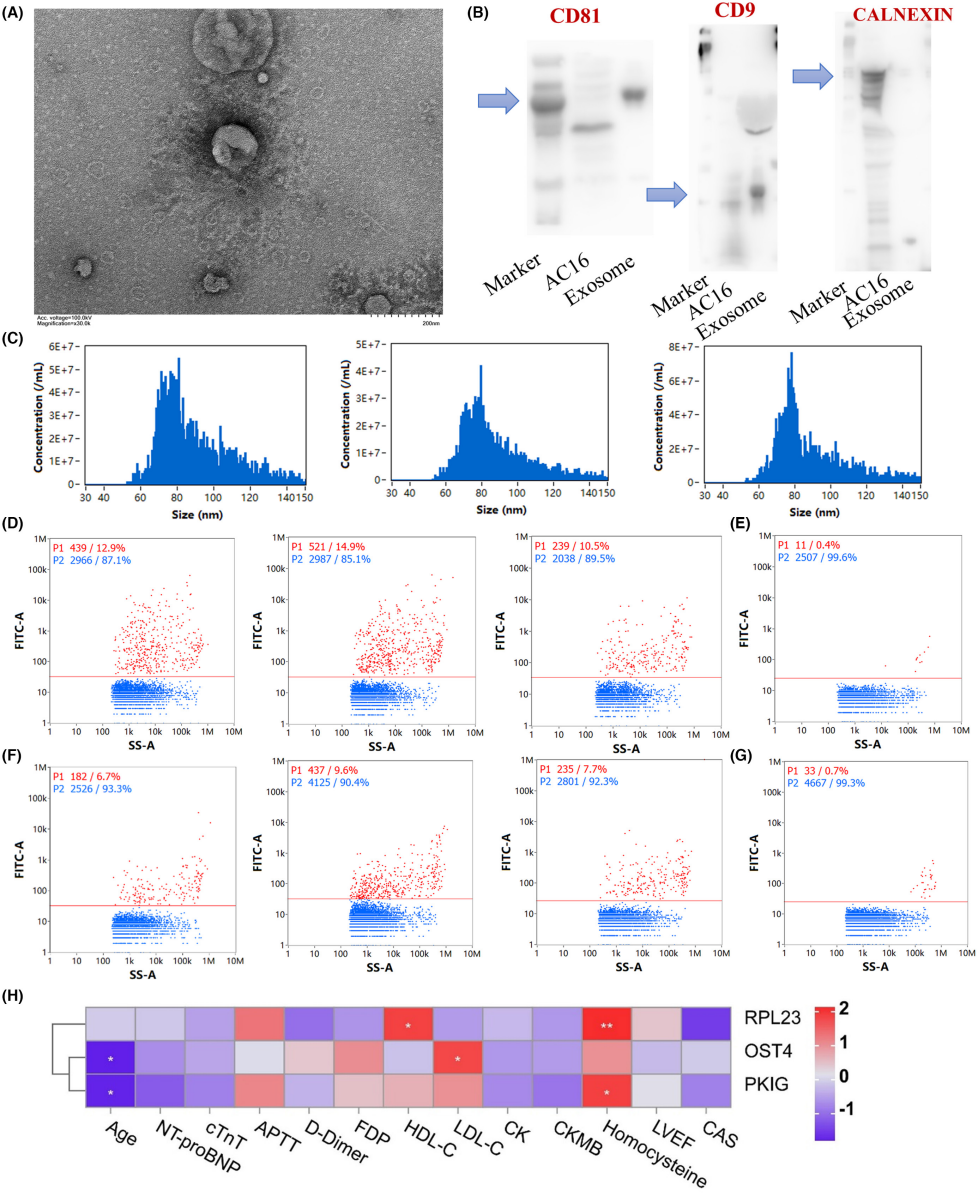
Exosome characteristics and clinical correlation analyses. (A) The cup‐shaped morphology of exosomes imaged by electron microscopy. (B) The characterization of exosomal biomarker proteins by western blot. (C) The particle size distribution detected by nano‐flow analysis. (D–G) The proportions of exosomal membrane proteins detected by nano‐flow analysis. (E, G) Negative controls. (H) Pearson correlation results between the gene expression level of early‐warning biomarkers within exosomes and clinical indicators in acute myocardial infarction.

The electron microscopy and nano‐flow cytometry analysis demonstrated that the captured vehicles had typical cup‐shaped and membrane‐enclosed characteristics of exosomes, with an average diameter of approximately 95.4 nm (Figure 6A,C). Western blotting revealed positive results for specific exosome proteins, including CD9, CD81, and TSG101 and showed no detection of the specific mitochondrial protein Calnexin (Figure 6B). The positive percentages of exosome‐related membrane proteins obtained from the nano‐flow cytometry analysis indicated an average percentage of 12.8 for CD9 (Figure 6D,E) and an average percentage of 8 for CD81 in total plasma exosomes (Figure 6F,G). These results confirmed the presence of exosome‐related membrane proteins in the captured vehicles, and the partial positive rate is likely due to the compromised partial vehicle‐membrane integrity during capture and detection. Since the mitochondrial diameter is >200 nm, and they cannot be encapsulated in exosomes, it can be concluded that the captured vehicles do not belong to large extracellular vehicles. Thus, these properties suggest that the captured vehicles are exosomes. Clinical correlation analysis revealed that *RPL23* was positively correlated with homocysteine and high‐density lipoprotein cholesterol (HDL‐C); while *OST4* was positively correlated with low‐density lipoprotein cholesterol (LDL‐C) and negatively correlated with age. As for *PKIG*, it was positively correlated with homocysteine negatively correlated with age (Figure 6H). The increased LDL‐C and homocysteine indicated the elevated severity of CAD. These results suggested that the *OST4* and *PKIG* were relevant with the CAD occurrence and the risk of disease progression.

### 3.6 Functional analysis and its correlation with early‐warning biomarkers

**FIGURE 7:**
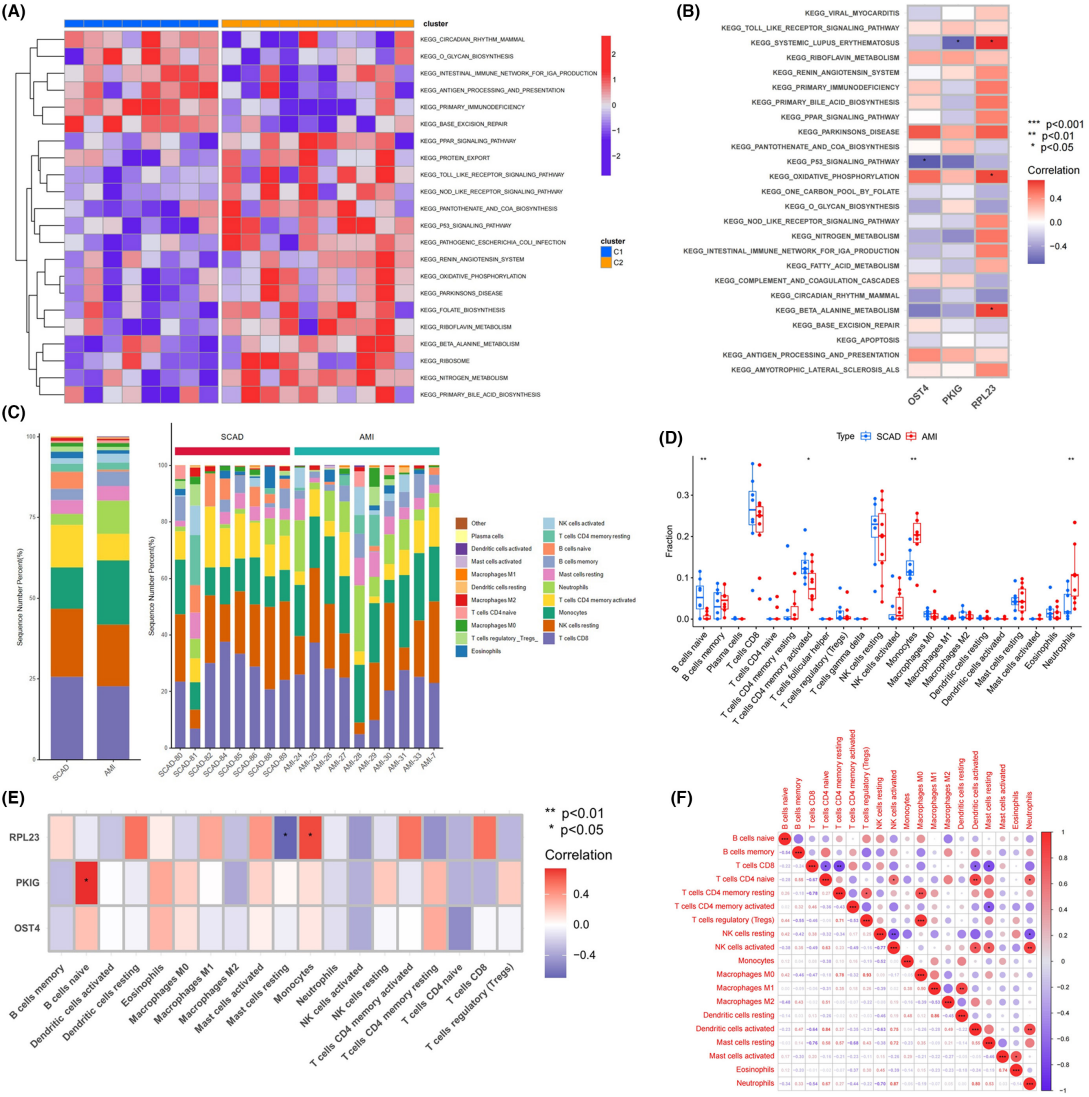
The potential relative fractions of different immune cell types reflected by exosomal RNA. (A) Heatmaps displaying differential enrichment scores in the acute myocardial infarction (AMI) samples compared with the stable coronary artery disease samples for the Kyoto Encyclopaedia of Genes and Genomes pathways using the single‐sample gene set enrichment analysis. (B) The association between early‐warning biomarkers and pathways enrichment scores in the AMI samples. (C) The deconvoluted specific‐cell proportion analysis of the plasma exosome sources (left: the average relative fractions of immune cell sources between groups; right: the relative fractions of immune cell sources in each sample). (D) The association between early‐warning biomarkers and immune cell types. (E) The association between early‐warning biomarkers and immune cells‐derived exosomes in AMI. (F) The synergistic effect for exosome release of immune cells in AMI.

Significant variations in the ssGSEA scores of the KEGG pathways between SCAD and AMI samples were observed (Figure 7A). In the AMI samples, a negative correlation was observed between the *OST4* expression and the ssGSEA scores of the p53 signalling and circadian rhythm pathways; conversely, a positive correlation was observed between the *RPL23* gene expression level and the oxidative phosphorylation and beta alanine metabolism pathways (Figure 7B). Furthermore, the results of GSEA showed that the top three *RPL23*‐promoted activation pathways according to the NES scores were in the processes of the adipocytokine signalling pathway; as for *OST4*, high expression *OST4* in the AMI samples could activate the metabolism‐related pathways including ascorbate and aldarate metabolism, drug metabolism cytochrome p450, steroid hormone biosynthesis and drug metabolism other enzymes; as for *PKIG*, high expression *PKIG* in the AMI samples could activate antigen processing and presentation, ascorbate and aldarate metabolism and drug metabolism other enzymes (Figure S1).

The relative fractions of diverse exosome sub‐populations from different immune cell compositions were estimated by using the CIBERSORT deconvolution method to clarify the biological significance linking the source‐tracking of plasma exosomes to immune cells (Figure 7C). In AMI samples, a positive correlation was observed between the relative fraction of monocyte‐derived exosomes and the *RPL23* gene expression level, as well as a negative correlation between the resting mast cells‐derived exosomes. A strong positive correlation was observed between the *PKIG* gene expression and naive B cells (Figure 7D). As anticipated, Figure 7E indicated that the relative fractions of monocyte‐derived and neutrophil‐derived exosomes were significantly higher, whereas the relative fractions of naive B cells and CD4 memory‐activated T cells were significantly lower in AMI samples than in SCAD samples. Furthermore, the fractions of CD8 T cells‐derived, activated memory CD4 T cells‐derived and resting NK cells‐derived exosomes were negatively correlated with other immune cells‐derived exosomes; whereby the fractions of naive CD4 T cells‐derived, resting memory CD4 T cells‐derived and activated NK cells‐derived exosomes were positively correlated with others (Figure 7F). These results indicated that distinct immune cells could have synergistic or antagonist effect on the process of exosomes release in AMI.

### 3.7 Early‐warning biomarkers mediated molecular patterns in AMI

**FIGURE 8:**
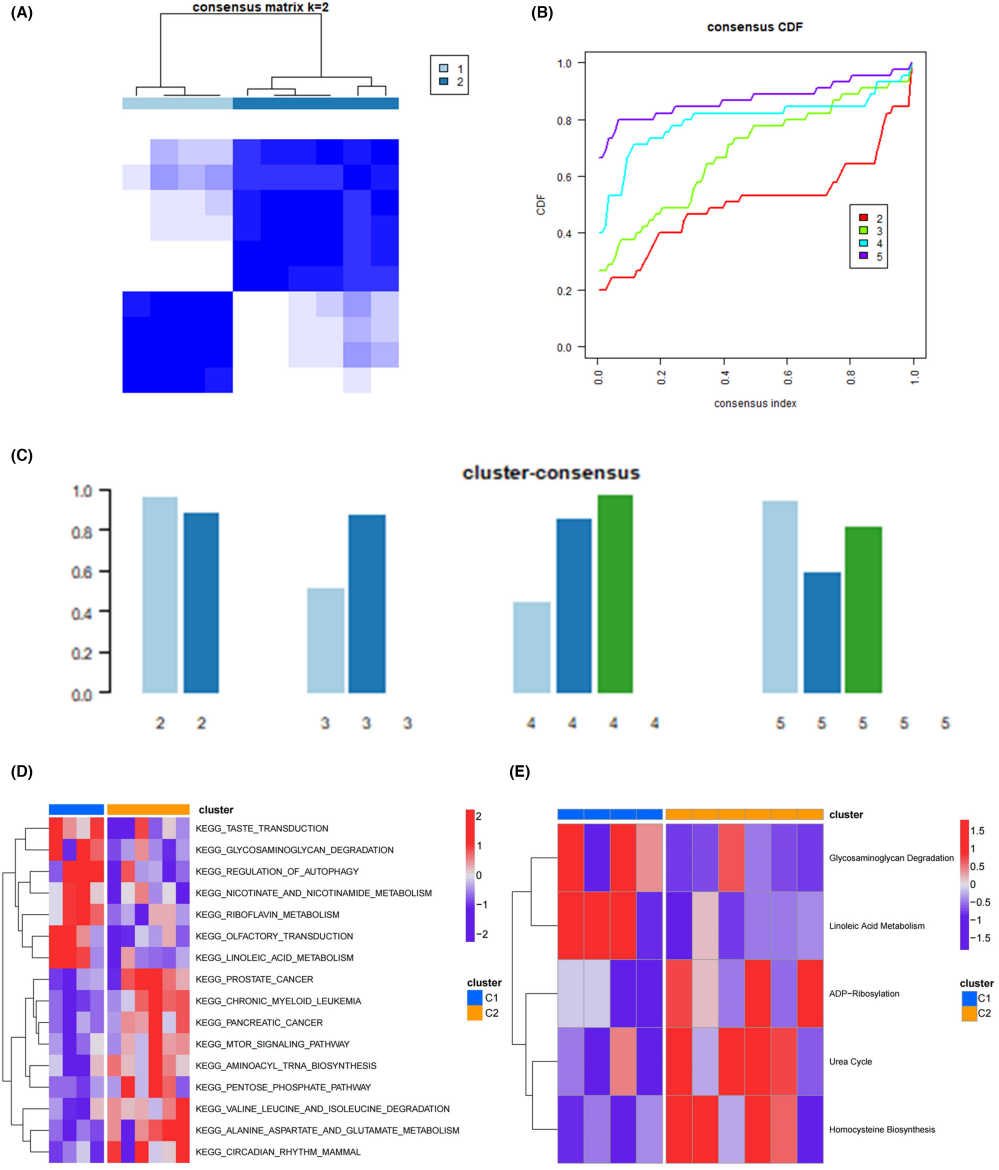
The landscape of the molecular cluster in the acute myocardial infarction samples. (A) Consensus matrix heatmap under *k* = 2 reflected optimal clustering. (B) Cumulative distribution curves under cluster counts of 2–5. (C) The consensus score for diverse clusters. (D) The enrichment differences of KEGG pathways between clusters. (E) The differential enrichments of metabolism‐relevant signatures between clusters.

The consensus score for each subtype was >0.8 while clustering the AMI samples into two subtypes,^11^ indicating a more classification (Figure 8A); this was corroborated by the genotyping results with *k* = 2, which yielded high stability from the consensus matrix and empirical cumulative distribution function curve (Figure 8B,C). Marked heterogeneities of the molecular function landscape among different subtypes were observed. The relative enrichment scores of folate biosynthesis, riboflavin metabolism, linoleic acid metabolism, hexose phosphate transport and peptidase activity were significantly higher in cluster‐2 than in cluster‐1, indicating that the metabolism function profiles varied considerably among patients with AMI (Figure 8D). As the various AMI clusters had heterogeneous metabolic preferences, we further compared the comprehensive metabolic‐related characteristics between the clusters. Differential analyses revealed that cluster‐2 exhibited higher enrichment in adenosine diphosphate‐ribosylation, urea cycle and homocysteine biosynthesis (Figure 8E).

## 4 DISCUSSION

A great variety of RNA species is present and maintained within exosomes due to the intact double‐layer membrane structure, making exosomes an excellent strategy for non‐invasive liquid biopsy. Research into exosomal long‐RNAs (mRNAs, circular RNAs, and long non‐coding RNAs) has been increasing, and in particular, mRNAs, the most abundant RNA species among exosomal long‐RNAs, have been found to have functional and clinical implications.^12^

In this study, the genetic signature of mRNA associated with heart exosomes in AMI was established and developed for early‐warning biomarkers of AMI occurrence. We found that a high proportion of the tissue‐specific genes from the heart, were also detected in plasma exosome samples. This might imply that the gene expression profiles of plasma exosomes carrying the parental tissues genes could potentially reflect the exosome's tissue of origin, as different tissues have distinct gene expression profiles, and the RNA‐sequencing data of plasma total exosomes could be used for the reverse source‐tracking analysis of parental tissues or cells abundances through the deconvolution method. The source‐tracking analysis suggested that plasma total exosomes were mainly derived from the spleen and whole blood cells, with minor contributions by the heart, possibly because the blood cell‐enriched blood serves as a reservoir of plasma total exosomes, and the spleen is closely associated with the haematopoietic functions and blood cell maturation. This similarity was reported by Lai et al. in the source‐tracking of heart exosomes in plasm samples, where again plasma total exosomes had relatively few cases derived from the heart tissue among adipose, bladder, brain, colon, oesophagus, kidney, liver, lung, muscle, nerve, pancreas, pituitary, skin, small intestine and stomach tissues.^8^ The GO analysis demonstrated that the heart exosomes genetic signature was enriched into heart development, cardiac function and cardiac response to stress and was not associated with other tissue‐related terms, confirming that the identified genetic signature was associated with the heart. As such, a genetic signature could reflect the genetic network within heart exosomes alterations of this genetic signature might precisely provide information about the CM injury conveyed by the heart exosomes.

In the early‐warning biomarkers panel, the *OST4*, *PKIG* and *RPL23* genes were identified and used to create a non‐invasive and straightforward nomogram model for accurately predicting the risk of CAD exacerbation. High‐sensitivity troponin is only concerned with AMI diagnosis rather than prediction and early warning. This nomogram can be used at the microscopic level as a supplementary tool for clinical indicators. Further research in a larger, more diverse population of patients with CAD patients is needed to validate this model, yet it still has guiding implications for early warning prior to disease deterioration.

*PKIG* is a gene that encodes an isoform of the protein kinase inhibitor of cyclic adenosine monophosphate‐dependent protein kinase and is significantly expressed in the heart.^13^ There is no evidence of an association between *PKIG* and CAD; however, a previous study reported that a single nucleotide polymorphism in the mice *PKIG* gene in the aortic root is associated with susceptibility to atherosclerosis, and *PKIG* might be a potential underlying factor.^14^ Herein, we observed strong positive correlations between the *PKIG* gene expression level and naive B cells. In the study by Xu et.al, following myocardial infarction and the resulting autophagy‐induced cell death and maturation arrest in bone marrow B cells due to the release of glucocorticoid, the number of bone marrow B cells significantly decreased, particularly those of the bone marrow‐derived naive B cell subset.^15^ Meanwhile, the inclusion of bone marrow‐derived naive B cells significantly reduced the infarct size and improved cardiac function.^15^ This might imply that the *PKIG* gene expression level potentially affects the worsened or improved states of patients with AMI patients states in the exosome's activities associated with naive B cells, and why the relative fractions of naive B cell‐derived exosomes observed in AMI samples were significantly lower than those observed in SCAD samples.

*RPL23* is a gene that encodes ribosomal protein L23 and its high gene expression level relates to unfavourable events in diverse cancer diseases.^16^, ^17^, ^18^ Herein, we demonstrated that the *RPL23* gene expression level was elevated in AMI exosome samples. Furthermore, our analysis indicated that *RPL23* was positively correlated with the fractions of monocyte‐derived exosomes and negatively correlated with the fractions of resting mast cell‐derived exosomes. A previous study reported that plasma monocyte‐derived exosomes could transmit monomeric C‐reactive protein which has pro‐inflammatory properties, to maintain the chronic inflammatory state in CAD.^19^ This suggests that *RPL23* might be involved in regulating the inflammatory response of patients with AMI in exosomal activities.

*OST4* is expressed at a relatively high level in heart tissues, encoding a small membrane which is a subunit of oligosaccharyltransferase and involved in N‐glycosylation catalysis of nascent polypeptides in the endoplasmic reticulum lumen.^20^ Dysregulation of the p53 signalling pathway has been associated with a series of adverse events in CAD, such as p53‐mediated CM apoptosis.^21^ The link between *OST4* and the p53 signalling pathway suggests that *OST4* in exosomes might interact with the p53 signalling pathway to affect the fate of CMs. Compared with the study of Song et al., which used the transcriptome data of peripheral blood mononuclear cells to screen gene‐related early warning markers,^1^ the indicators selected in our study can not only be used as early warning indicators of disease deterioration at the macroscopic level, but also accurately reflect the changes of genetic information inside myocardial cells in the state of myocardial infarction.

The molecular subgroups can only be detected from patients' transcriptomes which, in turn, map to distinctive clinical phenotypes through consensus clustering.^11^ Increasing studies demonstrate that the heterogeneity of disease molecules influences local cell function, clinical outcomes and therapy response.^22^, ^23^ For example, in an analysis of lower‐grade glioma samples, three immune subgroups were found to have different genetic alterations and lymphocyte signatures.^24^ Additionally, an exploration was performed into the classification of AMI based on early‐warning biomarker profiling to facilitate better stratification of patients with AMI responsive to clinical therapy. Our pathway enrichment showed that different AMI clusters had heterogeneous metabolism preferences. This might be explained by the fact that myocardial infarction primarily affects the heart; however, other organs such as the liver, could also sustain injury. It is thought that multiple glycosaminoglycans, particularly heparin, act as complement inhibitors and contribute to a positive therapeutic effect in AMI.^25^ Riboflavin has been reported to attenuate myocardial infarction in a mouse model through the lysine‐specific demethylase 1‐mediated interaction between phospholipid metabolism and histone methylation, and there is evidence that riboflavin intake decreases as CAD severity increases.^26^ These findings suggest that cluster‐1 patients could benefit from treatment targeting these pathways. Mammalian target of rapamycin (mTOR) is associated with CM apoptosis and death upon ischemia–reperfusion injury.^27^ mTOR inhibitions has been shown to reduce the infarction size and left ventricular remodelling following myocardial infarction in an animal model. The molecular drug targets of Qishenkeli that are relevant to coronary heart disease involve aminoacyl transfer RNA (tRNA) biosynthesis.^28^ Homocysteine might contribute to atherosclerosis by inducing oxidative damage of the vascular endothelium and a high serum homocysteine level reflects AMI severity.^29^ Targeting mTOR, aminoacyl tRNA, and homocysteine might represent a potential therapeutic vulnerability in cluster‐2 patients.

## 5 CONCLUSIONS

In conclusion, our study introduced a panel of three early‐warning biomarkers associated with heart exosome genetic signatures, which accurately described the genetic information of heart exosomes carrying AMI signals and provided new insights for the exosomes research in CAD progression and prevention. Nevertheless, there are certain limitations. It is yet to be established whether the altered gene expression levels of these early‐warning biomarkers within exosomes contribute to the promotion of CADs or the amelioration due to intercellular crosstalk. Besides, further research into the predictive nomogram and molecular classifier in a larger cohort of patients is necessary to determine their potential implications.

## AUTHOR CONTRIBUTIONS

**Xiaojun Jin:** Conceptualization (equal); data curation (equal); writing – original draft (lead). **Weifeng Xu:** Investigation (equal). **Qiaoping Wu:** Investigation (equal). **Chen Huang:** Data curation (equal). **Yongfei Song:** Investigation (equal). **Jiangfang Lian:** Conceptualization (equal).

## FUNDING INFORMATION

This work was supported by the National Natural Science Foundation of China (81870255), Zhejiang Provincial Science Foundation of China (LY21H020001, LQQ20H160001), Zhejiang Provincial Medicine & Healthcare technology project of China (2021KY306), Ningbo Municipal Science Foundation of China (2021J296) and Traditional Chinese Medicine Scientific Research Fund Project of Zhejiang Province (2021ZB263).

## CONFLICT OF INTEREST STATEMENT

The authors declare no conflict of interest.

## Supporting information


Figure S1.

Table S1.

Table S2.

Table S3.

## Data Availability

The datasets used and/or analysed during the current study are available from the corresponding author on reasonable request. The data that support the results of current study is available on Gene Expression Omnibus (GEO) websites.
