# Supplementary figures and images for: Detecting early‐warning biomarkers associated with heart‐exosome genetic‐signature for acute myocardial infarction: A source‐tracking study of exosome
Source: J Cell Mol Med. 2024 Apr 25;28(8):e18334. doi: 10.1111/jcmm.18334 (PMC11044819; doi:10.1111/jcmm.18334)

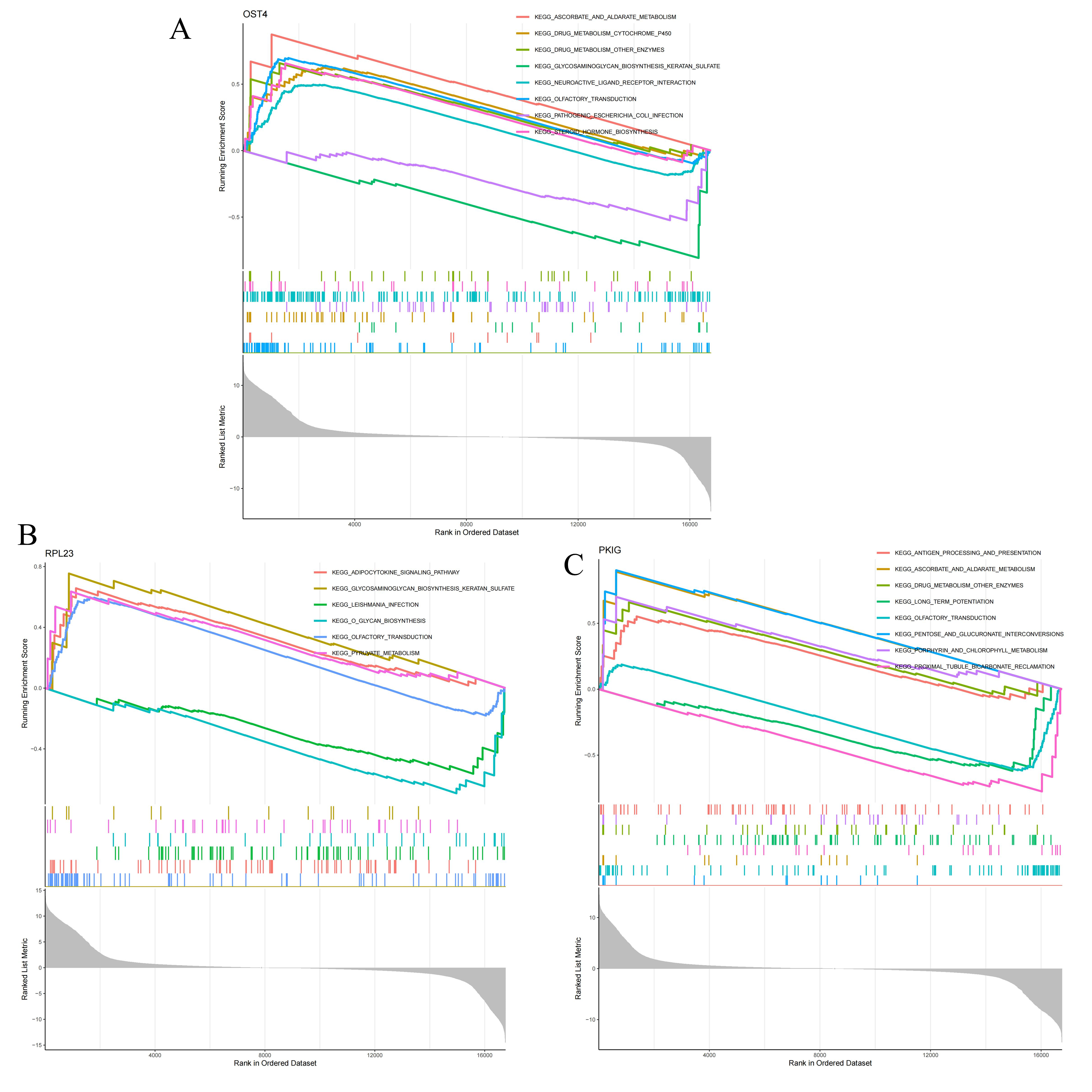

Supplement: Supplementary file 1 — Figure S1. [file JCMM-28-e18334-s003.tif]
